# Bovine papillomavirus E5 and E7 oncoproteins in naturally occurring tumors: are two better than one?

**DOI:** 10.1186/1750-9378-8-1

**Published:** 2013-01-09

**Authors:** Annunziata Corteggio, Gennaro Altamura, Franco Roperto, Giuseppe Borzacchiello

**Affiliations:** 1 Department of Pathology and Animal Health, University of Naples Federico II, Via Veterinaria, Napoli 1 80137, Italy

**Keywords:** Bovine papillomavirus, Cow, E5, E7, Equids, Papillomas, Sarcoid, Viral oncogenes

## Abstract

Bovine papillomaviruses (BPVs) are oncogenic DNA viruses, which mainly induce benign lesions of cutaneous and/or mucosal epithelia in cattle. Thirteen (BPV 1–13) different viral genotypes have been characterized so far. BPVs are usually species-specific but BPV 1/2 may also infect equids as well as buffaloes and bison and cause tumors in these species. BPV-induced benign lesions usually regress, however occasionally they develop into cancer particularly in the presence of environmental carcinogenic co-factors. The major transforming protein of BPV is E5, a very short hydrophobic, transmembrane protein with many oncogenic activities. E5 contributes to cell transformation through the activation of the cellular β receptor for the platelet-derived growth factor (PDGFβ-r), it also decreases cell surface expression of major histocompatibility complex class I (MHCI) causing viral escape from immunosurveillance, and plays a role in the inhibition of the intracellular communication by means of aberrant connexin expression. E7 is considered as a weak transforming gene, it synergies with E5 in cell transformation during cancer development. E7 expression correlates *in vivo* with the over-expression of β1-integrin, which plays a role in the regulation of keratinocytes proliferation and differentiation. Additionally, E7 is involved in cell-mediated immune responses leading to tumour rejection, in anoikis process by direct binding to p600, and in invasion process by upregulation of Matrix metalloproteinase1 (MMP-1) expression. Studies on the role of BPV E5 and E7 oncoproteins in naturally occurring tumours are of scientific value, as they may shed new light on the biological role of these two oncogenes in cell transformation.

## Introduction

Papillomaviruses (PVs) are small oncogenic DNA viruses infecting humans as well as different animal species, causing benign hyperproliferative lesions of both mucosal and cutaneous epithelia or malignant cancers. BPVs belong to the PV family and infection of bovines by BPVs is associated with the development of cutaneous papillomas, alimentary and urinary bladder tumours. BPVs are usually species-specific, and, even in experimental conditions, do not infect any host other than the natural one. However, BPV type 1 (BPV-1) and BPV type 2 (BPV-2) represent a well known case of cross-species infection since they also infect equids as well as buffaloes and bisons [1-4]. BPV-induced benign lesions usually regress, however occasionally they develop into cancer of both epithelial and mesenchymal origin. The major transforming protein of BPV is E5, a very small membrane-associated protein with potent biological activities [5]. The carcinogenic functions of E5 have been largely investigated *in vitro*, however many recent findings have highlighted the role of this oncoprotein also in naturally occurring oncogenesis.

BPV E7 is considered as a transforming gene although very little is known about E7-mediated mechanisms underlying carcinogenesis, so far. It has been hypothesized that BPV-E7 cooperates with E5 in cell transformation [6].

BPV has largely contributed as animal model to understand the genetics of PVs, the role of the early genes in cell transformation and still continues to do so due to very recent discoveries in naturally occurring equine and bovine tumours [7].

Furthermore, spontaneously arising BPV induced tumors are of interest not only in veterinary pathology but also from a comparative point of view. BPV E5 and E7 oncogenes have been studied at length up-to-date opening new scenarios about the possible role of these oncogenes in PV-induced cell transformation.

This review, will describe in brief the biological features of BPV E5 and E7 oncoproteins and will focus on the activity and the expression patterns of the two oncoproteins *in vivo* in naturally occurring bovine and equine tumours.

## BPV oncoproteins: general biological characteristics

All PVs have a circular double-stranded DNA genome. The genetic organisation of BPVs is similar to that of other PVs. The open reading frames (ORFs) are divided into early and late regions. The early region encodes non structural proteins from E1 to E7. The late region encodes two structural proteins: L1 and L2. In addition, a non-coding long control region provides the cis-regulatory elements necessary for viral replication and transcription [8]. The transforming activities of BPVs are due to three early viral genes: E5, E6, and E7. The specific contribution of each oncogene in transforming process varies between different BPVs. Some BPVs (BPV-3,-4,-6) lack the E6 gene, although they are able to give a successful infection and to induce tumours [9].

Despite the clear evidence that E6 is an efficient transforming protein *in vitro*[10,11], the activity of E6 *in vivo* in naturally occurring tumours still remains uncertain and needs to be investigated.

The growing body of recent studies *in vivo* highlights the central role of E5 and E7 expression/activity in tumour development strengthening the focus of this review.

### E5 oncogene

The major transforming protein of BPV is E5, a very small, membrane associated protein with different biological activities and critical to the transformation process [5,12,13]. BPV-1 E5 is the shortest oncoprotein yet characterized being only 44-aminoacid long [12,14,15]. Genetic and biochemical studies have revealed that the E5 protein can be divided into two distinct domains: an amino-terminal domain (residues 1–32), consisting of strongly hydrophobic aminoacid residues and a single hydrophilic aminoacid, and an hydrophilic carboxyl-terminal domain (residues 33–44). The hydrophobic domain is presumed to mediate the association with cellular membranes and to function as a signal-anchor domain whereas the hydrophilic domain is believed to interact with important cellular regulatory proteins [16]. In accordance with its predicted hydrophobic composition, the E5 protein spontaneously localizes in the membranous compartment where it is present as a dimer. The C-terminal domain is involved in E5 dimer and oligomer formation. Oligomerization appears to be essential for the transforming activity [12,17]. E5 accumulation in the late (trans-) Golgi apparatus (GA) is critical for its mitogenic signalling. Retention of E5 in the ER and early (cis-) Golgi causes loss of transformation activity [18]. Mutagenesis analysis underscore the critical role of individual aminoacid residues in mediating E5 biological activities. Several studies indicate that the hydrophobic nature of BPV E5 and a handful of important aminoacidic residues confer transforming activity [17]. These essential aminoacids are the most well conserved among related papillomaviruses [19].

E5 induces cell transformation through the activation and/or impairing of several intracellular targets involved in cell proliferation and survival [5].

### E7 oncogene

The BPV-1 E7 oncogene is a zinc binding protein 127 aminoacids long. E7 does not show a strong independent transforming activity, but when it is coexpressed with E5, its transformation capacity is pronouncedly increased [6]. Mutants lacking the E7 open reading frame are still able to induce transformation but with lower efficiency [20]. It is not clear how BPV-1 E7 contributes to cell transformation as this oncoprotein, in contrast to HPV-E7, lacks the LXCXE motif that mediates the binding to the pRB family proteins [21]. It has been reported that the transformation activity of BPV-1 E7 is mediated, at least in part, by its ability to bind p600. The binding between BPV-1 E7 and p600 contributes to cell transformation by inhibiting the anoikis, a type of apoptosis, which is commonly encountered in cancer cells, allowing them to survive in the absence of normal cell-matrix interactions [22]. In addition, BPV-1 E7 seems to sensitize the cells to TNF-induced apoptosis [23,24]. On the contrary E7 is the major transforming protein of BPV-4 as defined in *in vitro* systems [25,26]. BPV-4 E7 contains two Cys-X-X-Cys motif and the pRb-binding domain, both are critical for its transforming activity [25].

Most of the studies on BPVs have been performed in *in vitro* systems where the biological characteristics and functions of BPV oncoproteins have been largely investigated, however the functions of BPV oncoproteins *in vivo* are less understood. Recent insights into E5 and E7 biological activities strenghten the pivotal role of these oncoproteins in cell transformation in naturally occurring tumours.

## BPV E5 and E7 in naturally occurring tumours

BPV infection is associated to cutaneous papillomas, alimentary fibropapillomas and urinary bladder tumours in cattle and to sarcoids in equids.

### Bovine cutaneous papillomas

**Figure 1:**
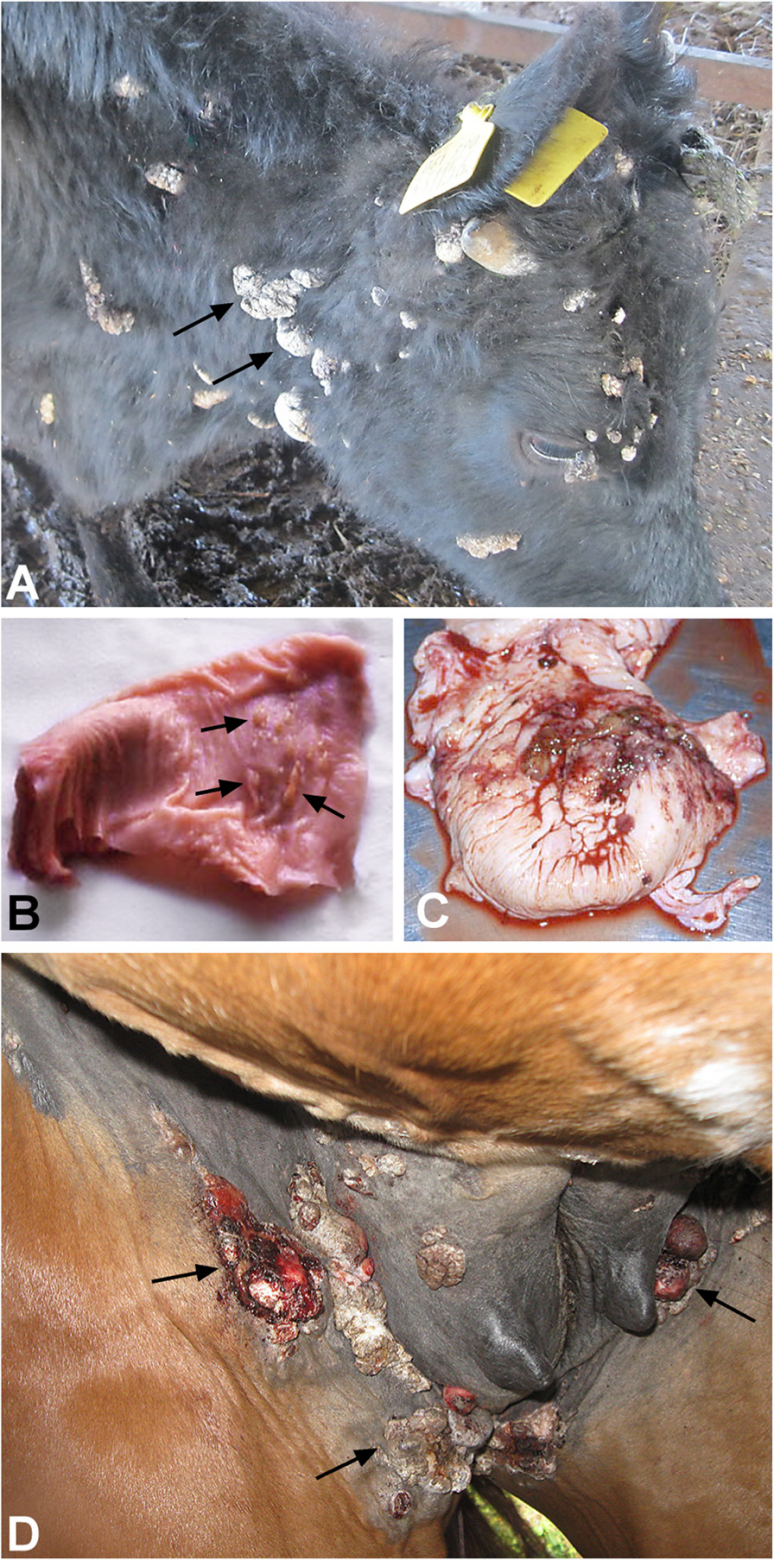
**Examples of BPV induced tumours in its natural host, bovine, and in its heterologous host, horse. A.** Persistent papillomatosis of the skin in a cow. **B.** Diffuse papillomatosis of the oesophagus in a cows , the arrows indicate the papillomas. **C.** Multiple haemangiosarcomas and urothelial carcinoma of the urinary bladder in a cows. **D.** Multiple nodular sarcoids in the paragenital and mammary region in a horse.

Cutaneous papillomatosis is characterized by the presence of multiple benign exophitic proliferations of the epithelia (Figure 1A). At least 11 different types of BPVs have been identified in association with cutaneous papillomas and fibropapillomas (BPV-1, BPV-2, BPV-5, BPV-6, BPV-7, BPV-8, BPV-9, BPV-10, BPV-11, BPV-13) [1,27,28]. When BPV-infection of the skin causes also the transformation of dermal fibroblasts and epithelial acanthosis, the lesion is defined as fibropapilloma. Papillomas and fibropapillomas may localize in different body sites in cattle [29].

During papilloma formation, the expression of BPV early and late genes is strictly regulated. The virus initially infects the basal keratinocytes. The early genes are expressed in undifferentiated basal and suprabasal layers, determining the re-entry of spinous cells into the cell cycle and amplification of viral DNAs in a process termed vegetative viral replication [4]. The distribution of BPV E5 and E7 proteins have been studied in bovine fibropapillomas.

In bovine fibropapillomas two sites of E5 expression have been identified within the epithelium: the cytoplasm of basal cells and superficial granular cell layer. High concentrations have been noted for E5 protein in the differentiated tumour keratinocytes. The granular staining pattern is associated with the sites of viral capsid synthesis [30]. The expression pattern of E5 and its localization in the lesions are suggestive for E5 actively contributing to successfull BPV (de novo) infection.

The localization in the basal layers indicates that E5 may have a role during the early stages of viral infection and tumour development and it may lead to sustained cell proliferation to favor virus-infected cells. Its presence in the more differentiated layers suggests an involvement of E5 in the late stages of the viral life cycle [6].

Interestingly, BPV-1 E5 has also been found to be expressed in cutaneous fibropapillomas from water buffaloes [2]. This observation suggests that the oncoprotein plays a role in cutaneous fibropapillomas development independently of the infected species.

Typically E5 has been detected mostly within the cytoplasm of infected epithelial and fibroblastic cells, furthermore, some of the cells showed accentuated staining on one side of the nucleus, in a perinuclear region, consistent with the localization in the Golgi apparatus (GA) [30].

In E5 expressing fibropapillomas alterations in Connexin26 (Cx26) expression levels and in its intracellular localization have been demonstrated [31]. Interestingly, Cx26, a component of the skin connexons, colocalizes with E5 in a juxtanuclear region. This observation supports the hypothesis for a possible role of E5 in alteration of the Gap junctions intercellular communication mediated by Cx26 dysregulation. It is likely that the down-regulation of the gap junctions by E5 makes the transformed cells refractory to growth inhibitory signals from neighbouring cells [31]. It is worth noting that cutaneous fibropapillomas expressing E5 show hyperactivation of the PDGFβR and its downstream transforming signals, suggesting a possible role of this cell signaling in lesion development [32].

BPV-E7 is expressed in the cytoplasm and nucleus of the spinous layers where vegetative BPV amplification is known to occur [6]. In bovine fibropapillomas E7 is localized in the basal layers of neoplastic epithelium where it colocalizes with E5. The co-expression of both proteins in the basal layer of fibropapillomas suggests that they may cooperate in controlling cell proliferation, and it is possible that E7 expression serves to modulate the actions of E5 on epithelial cells [6].

Remarkably, BPV-1 E7 expression in fibropapillomas correlates with the over-expression and the aberrant expression of β1-integrin and PCNA in suprabasal epithelial cells. β1-integrin is a member of the integrin family, which has a role in the regulation of keratinocytes proliferation and differentiation. The observation that the distribution of β1-integrin within the fibropapilloma was reminiscent of the distribution of BPV-1 E7, indicates that E7 is implicated in the induction of vegetative DNA amplification in keratinocytes of the spinous layer [33].

### Bovine fibropapillomas of the upper gastrointestinal tract

The mucosal epithelium of the alimentary canal of cattle may be infected by BPV type 4 where it induces fibropapillomas (Figure 1B) [34]. Epithelial papillomas of the upper gastrointestinal (GI) tract can develop at every site from the mouth to the rumen. Histologically, they have the same features of benign fibropapillomas where the active viral replication results in inclusion bodies visible in the nuclei of infected cells [35,36]. Fibropapillomas regress in healthy immunocompetent animals due to an efficient cell-mediated immune response. Cattle feeding on bracken fern develop upper alimentary canal cancers as a result of an interactions between the virus and the toxic and carcinogenic substances contained in bracken fern [8,37].

The transforming activities of BPV-4 is due to the viral genes E5 and E7. BPV-4 E5 encodes a small hydrophobic polypeptide similar in its length and amino acid composition to the BPV-1 E5 polypeptide (see above). BPV-4 E7 unlike BPV-1 E7 possesses the canonical Cys-X-X-Cys motif and the pRb interaction domain, that is crucial for its transforming ability [25]. Both proteins are expressed in virally induced alimentary canal tumours: E5 is mostly expressed in early development stages, on the contrary E7 is produced throughout all stages of the fibropapilloma, suggesting its pivotal role in the maintenance of the proliferative state of the epithelia and its necessary role in cell transformation [38].

BPV-4 E5 is expressed in fibropapillomas of the alimentary canal where it localizes in the basal layers [38]. Following viral infection of basal keratinocyte, E5 induces down-regulation of surface MHCI expression. This is an important observation for the notion that E5 has a role in the establishment of BPV infection in fibropapillomas of the alimentary canal. E5 may contribute to BPV escape from immunosurveillance through the prevention of viral antigen presentation to immune effector cells [39].

In alimentary carcinomas E7 was uniformly detected within the nuclei of basal and suprabasal cells and within the cytoplasm of differentiated spinous and granular cells, suggesting that this different intracellular localization might correspond to different functions [6].

The more extensive analysis of vaccination and immune response in relation to BPV-4 induced tumours was indicative for the possibility that E7 has an important role in cell-mediated immune responses leading to tumour rejection [40,41]. Experimental rejection of BPV-4 papillomas has been achieved by immunizing cattle with the viral protein E7, and the regressing papillomas showed infiltrates of immune cells similar to those observed in naturally regressing tumours.

### Bovine urinary bladder tumours

Tumours of the urinary bladder in cattle are of epithelial and mesenchymal origin (Figure 1C) [42,43] and commonly associated with chronic enzootic haematuria (CEH), a syndrome caused by prolonged ingestion of bracken fern [8]. The involvement of BPV-1/2 in urinary bladder carcinogenesis is well recognized and the synergistic relationship between the virus and carcinogens from the fern has been experimentally reproduced [37].

The expression, localization and oncogenic activity of BPV E5 has been largely studied in BPV induced urinary bladder tumours. BPV E5 is expressed in the cytoplasm of both basal and suprabasal transformed urothelial cells. Expression of this oncoprotein is also seen in neoplastic blood vessels. E5 is found to be expressed intracytoplasmically with a typical juxtanuclear pattern suggesting Golgi localization [44-46]. This observation has been made on samples from different countries (Italy, Romania, Portugal, Brazil), confirming the specific localization of E5 protein *in vivo *[44,47,48]. The finding of E5 expression only in cancer samples but not in normal tissue suggests that the virus is involved in neoplastic processes and required for it [46]. In addition, intratumoral colocalization of E5 and E7 is noted, suggesting that cell transformation is triggered by these oncoproteins in a synergistic manner [49,50].

The molecular mechanism by which E5 induces cell transformation lies in its binding to, and activation of the PDGFβ-r, which is reflected by a potentiation of the mitogenic response [51]. Interestingly, Borzacchiello et al [52], have shown that in naturally occurring bovine urinary bladder tumours BPV E5 is able to physically interact with the PDGFβ-r and that the receptor is more activated in cancer, confirming the role of the protein complex in cancer development. Moreover, the binding of the E5 and the PDGFβ-r induces the activation of different signal transduction pathways: the PDGFβ-r and PI3K physically interact such as PDGFβ-r and the Grb2-Sos complex. PI3K-Akt and Grb2-Sos-Mek-Erk signals are all potentiated in cancer, but the levels of phosphorylated (activated) Erk and Mek proteins are not significantly overexpressed [53-55]. Cattle suffering from urinary bladder cancer harbour BPV DNA in blood cells where recently E5 expression has been demonstrated, supporting the hypothesis that blood may constitute a reservoir of the virus and suggesting a possible biological activity of BPV in the blood of these animals [56,57].

It has been suggested that there is a possible direct or indirect role of E5 oncoprotein in the inhibition of intracellular communication in naturally occurring cancer. In fact, the expression of Cx43 was shown to be altered in different bovine urothelial neoplastic lesions expressing E5 oncoprotein. The expression of Cx43 was reduced in carcinoma in situ (CIS) samples, and was lost in neoplastic invasive urothelial cells suggesting a progressive loss of Cx43 during cancer development [58].

It is worth noting that the BPV-2 E5 oncoprotein has many biological characteristics similar to the viral HIV Negative Regulatory Factor (Nef) protein, which is involved in iron metabolism via the Ferritin heavy chain(HFE) [59]. The ferritin heavy chain is upregulated in E5 positive urothelial neoplasia [60]. The same samples express hepcidin (F Roperto, personal observations), an antimicrobial hormone known to be a key regulator in iron homeostasis. This observation suggests a possible involvement of E5 in iron homeostasis during the development of urothelial tumours.

During cell transformation, different cellular pathways are activated, among these the arachidonic acid metabolism seems to have an important role in tumour development [61]. E5 activates arachidonic acid metabolism independently of PDGFβ-r activation [62]. Interestingly, in spontaneously arising bovine urinary bladder carcinomas the cyclooxygenase enzymes are overexpressed, confirming a possible role of the E5 in the upregulation of arachidonic acid metabolism also *in vivo *[45].

Finally E5-expressing bovine urinary bladder tumours show activated calpain 3, suggesting a possible involvement of this protein in urothelial carcinogenesis [63]. It has been suggested that Calpain 3 plays a pro-apoptotic role in cancer development [64].

Interestingly, BPV-2 E5 has also been found to be expressed in urinary bladder tumours of water buffaloes [65]. This observation thus suggests that also in this case, the oncoprotein is involved in BPV-induced carcinogenesis independently of the infected species.

Finally BPV-2 E5 was found to be expressed and physically interacted with PDGFβ-r in the uterine and chorionic epithelium of the placenta in pregnant cows suffering from naturally occurring papillomavirus-associated urothelial bladder tumours. It is possible that E5 action on PDGFβ-r is involved in organogenesis and neo-angiogenesis rather than in cell transformation during pregnancy [66].

### Equine sarcoid

BPV is the only known case among PVs to cross infect other species. BPV-1/2 are able to infect equids inducing a common dermatological neoplasm called sarcoid. There are different clinical presentations of sarcoids (nodular, occult, fibroblastic, verrucous, malevolent and mixed, which is a combination of several of these types) (Figure 1D), but histologically they have all the same features: dermal proliferation of spindle-shaped fibroblasts features forming whorls and epidermal hyperplasia [1,67].

In sarcoids the E5 oncogene is transcriptionally active and the protein is expressed in the neoplastic fibroblasts and overlying hyperplastic epidermis where the virus completes its life cycle producing virions particles [68-71]. The levels of E5 m-RNA are higher in the nodular type of sarcoids as compared with the other clinical types, although a recent report indicates that the highest levels of E5 DNA are found in the lesions of horses suffering from an aggressive form of disease i.e. multiple, rapidly growing tumours of various type [72,73]. These data provide all evidence of active BPV infection in sarcoids supporting the role of E5 in cell transformation. Interestingly, sequence analysis of the E5 open reading frame of sarcoid-associated BPV revealed several unique DNA sequence variants, some of which are associated with increased codon usage [74,75]. E5 sequence variations can influence the cellular location and function of the oncoprotein and are contributing factors to the pathogenesis of the disease. Sarcoid lesions co-express BPV E5 and E7 oncoproteins in neoplastic fibroblasts. E5 is localized in the cytoplasm in a juxtanuclear position, whereas E7 is found expressed in the cytoplasm and nuclei; the co-expression of E5 and E7, suggests that these molecules may co-operate also in sarcoid development. The role of E5 in naturally occurring sarcoids has been largely investigated: E5 is found in molecular complex with the phosphorylated PDGFβ-r (pPDGFβ-r) in sarcoids and the high levels of pPDGFβ-r activate the PI3-K-Akt pathway and Grb2-Sos-Mek-Erk pathway (G Borzacchiello, personal observations) such as in bovine tumours [76]. It is therefore reasonable to assume that E5 can activate the same signal transduction pathways in bovine and equine species via the activation of the PDGFβ-r.

E5 is also found transcriptionally active in blood cells from sarcoid affected horses, thus suggesting a possible contribution to virus spread. The BPV genomes and, more importantly, the expression of E5 in equine inflammatory skin conditions suggest that BPV is also involved in this type of disease [77].

Very little is known about the carcinogenic role of E7 in equine sarcoids, but it has recently been shown that the interaction between the 600-kDa retinoblastoma protein-associated factor (p600) and BPV E7, described *in vitro* in cultured cells, takes place also *in vivo *[78]. Furthermore, intracellular signals involved in p600 functional activity are not overexpressed, suggesting a different functional activity of p600 in naturally occurring carcinogenesis. It has also recently showed that E7 may contribute to upregulate the expression of MMP-1 as suggested by a previous *in vivo* study [79,80]. These findings suggest that E7 may contribute to sarcoid cell invasion process through upregulation of MMP.

## Conclusions

PVs induced cell transformation is paradigmatically based on the different contribution of the three viral oncogenes E5, E6, and E7 affecting multiple cellular pathways.

Most of the studies about PV induced oncogenesis and the role of the viral oncogenes have firstly been recognized in the BPV model system and later validated for HPV.

**Figure 2:**
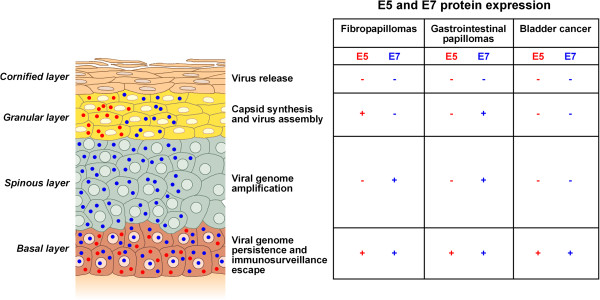
**E5 and E7 can improve BPV activity by controlling the viral replication and persistence, and virus assembly.** A schematic view of the expression of the BPV E5 and E7 genes in epithelium. BPV gene expression is tightly regulated and strictly linked to epithelial differentiation. In BPV naturally occurring epithelial tumours E5 and E7 colocalize in the basal layer. The expression of E5 and E7 in the basal layers of the epithelium, would lead to sustained cell proliferation to favour virus-infected cells. E5 also could contribute to a successful BPV infection establishment and persistence by inducing evasion of immune surveillance. In cutaneous fibropapillomas E7 is also expressed in the spinous layers suggesting its implications in viral DNA amplification, instead E5 is expressed in more differentiated granular layers suggesting its involvement in the late stages of virus life cycle (capsid synthesis). In fibropapillomas of the upper gastrointestinal tract E5 is expressed only in the basal layers, moreover E7 is expressed also in more differentiated spinous and granular layers suggesting its involvement in viral DNA amplification and in the late stages of virus life cycle. In bovine urinary bladder cancer E5 and E7 are expressed only in the basal layers.

The expression and functions of BPV E5 and E7 oncoproteins have been largely investigated in naturally occurring tumours (Figure 2) with E5 being the major oncogene. Recent studies about the functions of E5 *in vivo* have on the one hand confirmed *in vitro* studies, on the other hand have discovered new aspects.

**Figure 3:**
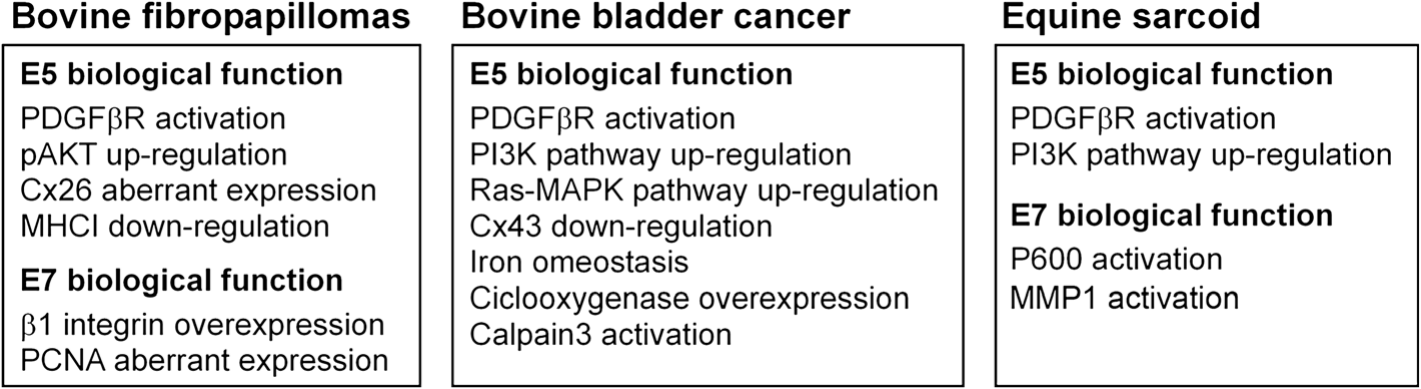
Schematic representation of cellular events mediated by BPV E5 and E7 responsible of cell transformation in naturally occurring tumours.

The role of BPV E7 in cellular transformation has been less investigated. To date, *in vivo* studies suggest a synergism between E5 and E7: they cooperate in the BPV infection establishment and persistence as well as in controlling cell proliferation and transformation processes (Figure 3), suggesting that “two are better than one” in naturally occurrying carcinogenesis.

Finally, *in vivo* studies about the expression and functions of BPV oncoproteins in experimentally PV-induced tumours are still relevant to better understand the oncogenic potential of the virus and to gain new insights into the interplay between PVs oncogenes.

## Competing interests

The authors declared that they have no competing interests.

None of the authors of this paper has financial or personal relationship with other people or organizations that could innappropriately influence or bias the content of the paper.

## Authors’ contributions

All authors have contributed equally to the paper. All authors read and approved the final manuscript.
